# Fragile X-Associated Tremor/Ataxia Syndrome (FXTAS): A Gender Perspective

**DOI:** 10.3390/jcm11041002

**Published:** 2022-02-15

**Authors:** Daniele Orsucci, Lucia Lorenzetti, Fulvia Baldinotti, Andrea Rossi, Edoardo Vitolo, Fabio Luigi Gheri, Alessandro Napolitano, Giancarlo Tintori, Marco Vista

**Affiliations:** 1 Unit of Neurology, San Luca Hospital, Via Lippi-Francesconi, 55100 Lucca, Italy; marco.vista@usl.nordovest.toscana.it; 2 Unit of Internal Medicine, Santa Croce Hospital, 55032 Castelnuovo Garfagnana, Lucca, Italy; lucia.lorenzetti@usl.nordovest.toscana.it (L.L.); edoardo.vitolo@usl.nordovest.toscana.it (E.V.); fabioluigi.gheri@usl.nordovest.toscana.it (F.L.G.); giancarlo.tintori@usl.nordovest.toscana.it (G.T.); 3 Laboratory of Molecular Genetics, University Hospital of Pisa, 56126 Pisa, Italy; fulvia.baldinotti@unipi.it; 4 Medical Affairs and Scientific Communications, 1260 Nyon, Switzerland; molezzano.1@libero.it; 5 Unit of Neurology, NOA Hospital, 54100 Massa, Italy; alessandro.napolitano@usl.nordovest.toscana.it

**Keywords:** ataxia, episodic ataxias, Fragile X, FXS, FXTAS, paroxysmal movement disorders, trinucleotide repeat diseases

## Abstract

Although larger trinucleotide expansions give rise to a neurodevelopmental disorder called fragile X syndrome, fragile X-associated tremor/ataxia syndrome (FXTAS) is a late-onset neurodegenerative disorder caused by a “premutation” (55–200 CGG repeats) in the *FMR1* gene. FXTAS is one of the more common single-gene forms of late-onset ataxia and tremor that may have a more complex development in women, with atypical presentations. After a brief presentation of the atypical case of an Italian woman with FXTAS, who had several paroxysmal episodes suggestive of acute cerebellar and/or brainstem dysfunction, this article will revise the phenotype of FXTAS in women. Especially in females, FXTAS has a broad spectrum of symptoms, ranging from relatively severe diseases in mid-adulthood to mild cases beginning in later life. Female FXTAS and male FXTAS have a different symptomatic spectrum, and studies on the fragile X premutation should be conducted separately on women or men. Hopefully, a better understanding of the molecular processes involved in the polymorphic features of FXTAS will lead to more specific and effective therapies for this complex disorder.

## 1. Introduction

Fragile X-associated tremor/ataxia syndrome (FXTAS) is a late-onset neurodegenerative disorder caused by a “premutation” (55–200 CGG repeats) in the *FMR1* gene at Xq27.3. Larger trinucleotide expansions (>200 CGG repeats) lead to a neurodevelopmental disorder called fragile X syndrome (FXS) [1], the most common cause of inherited intellectual disability and autism spectrum disorders.

FXS is the most frequent form of hereditary learning difficulty, estimated to affect one in 2500–7000 males and one in 2500–11,000 females [2]. Most males with FXS have an intellectual disability, mainly in the autism spectrum, and behavioral symptoms including anxiety and attention deficit. The mild to severe symptoms in females with FXS include learning difficulties and relationship difficulties [2].

FXS is thought to be a loss-of-function genetic condition. In contrast, the “premutation” leads to excessive levels of *FMR1* messenger RNA (mRNA) that causes toxicity. Repeat-associated translation from antisense CCG repeats generate novel proteins that accumulate in ubiquitinated inclusions in FXTAS patients [3].

The European Fragile X Network (EFXN) has proposed that “Fragile X Premutation Associated Conditions” (FXPAC) should be used as a coded term covering any condition linked to the fragile X premutation, including fragile X-associated primary ovarian insufficiency (FXPOI), fragile X-associated tremor/ataxia syndrome (FXTAS), and other less defined conditions presenting with a wide range of neuropsychiatric symptoms [2]. Obviously, there is great overlap between these clinical pictures.

FXTAS clinical features usually begin in subjects aged above 50 years with kinetic tremor, that is not always severe enough to be detectable, followed by cerebellar ataxia [4]. The term “cerebellar ataxias” includes a wide spectrum of neurological diseases and is characterized by imbalance (e.g., truncal ataxia, gait ataxia) or incoordination of a limb while executing a task (dysmetria). Ataxia is usually caused by a dysfunction of the cerebellum or its connections. Some patients with peripheral sensory disorders can also have ataxia. A neurological examination allows a correct distinction between the two conditions. Furthermore, cerebellar ataxia is frequently associated with other signs of cerebellar dysfunction, including abnormal eye movements, dysmetria, dysarthria, dysphagia, and kinetic tremor.

**Figure 1:**
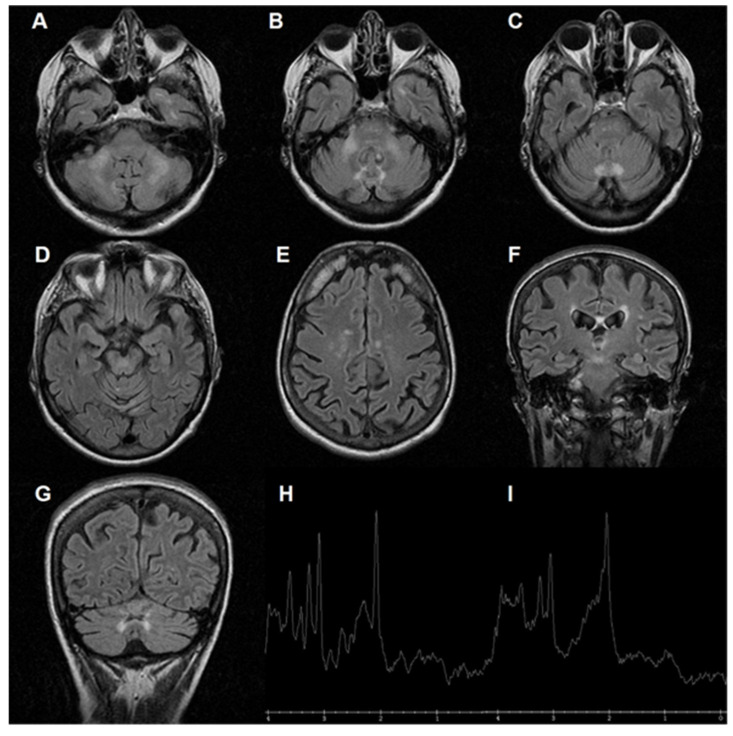
MRI of a 75-year-old woman with FXTAS. This patient was repeatedly hospitalized because of rare spells characterized by vomiting, dysarthria, dizziness, acute quadriparesis with bilateral Babinski sign, and consciousness impairment (once including coma requiring intubation). These attacks began when the patient was 71 years and typically lasted for a few days, fully recovering in about one week. She was having about one episode per year. The patient’s coexisting conditions include hypertension and well-compensated ischemic heart disease. She does not suffer from migraines. One of her grandsons was diagnosed with psychomotor retardation due to FXS. Interictal neurological examination was normal except for mild axial ataxia and postural tremor. The Mini-Mental State Examination revealed mild cognitive impairment (scored 20.7/30). No epileptic spikes were observed on the electroencephalogram (EEG). Laboratory assays were irrelevant. Echocardiography, repeated Holter ECG registrations and cardiac loop recording were normal. Electroneurography was normal. Brain CT angiography and neurosonological examination were repeatedly normal. She met the diagnostic criteria for “definite” FXTAS. Genetic testing (based on polymerase chain reaction techniques) confirmed this diagnosis, showing a normal allele with 22 CGG repeats and a “premutated” allele with 91 trinucleotides in the *FMR1* gene. (**A**–**C**) Axial fluid-attenuated inversion recovery (FLAIR) images showing increased signal in the middle cerebellar peduncles (“Middle Cerebellar Peduncle Sign”), cerebellar hemispheres, and vermis. Pontine pathology is evident in (**C**). (**D**,**E**) Axial FLAIR images reveal increased signals within the midbrain (**D**) and hemispheric cerebral white matter (**E**). (**F**,**G**) Coronal FLAIR images confirm periventricular, midbrain (**F**), and cerebellar (**G**) white matter disease. (**H**,**I**) Normal MRI spectroscopy of the cerebellum and vermis.

Older age has been associated with a significant increase in the prevalence of tremor and ataxia in male “premutation” carriers (i.e., the prevalence is 17% in patients aged between 50 and 59 years, and 75% in patients aged between 80 and 89 years) [1]. Parkinsonism, neuropathy, and dysautonomia have also been detected in patients with FXTAS. Memory and executive functions may be impaired (mildly in females) [1]. Females can be affected by FXTAS less frequently because they have a second X chromosome not carrying the premutation (females have two X chromosomes and, therefore, their probability of being a carrier of a premutated X is doubled). It has been estimated that about 40% of male and 16% of female carriers develop FXTAS. However, the premutation can occur in less than 1 in 200 women and 1 in 400 men, leading to a more balanced gender ratio [4]. MRI has shown that FXTAS can cause brain atrophy and white matter disease, usually in the corpus callosum, in the periventricular regions and especially in the middle cerebellar peduncles (i.e., “Middle Cerebellar Peduncle Sign”) [1] (Figure 1).

FXTAS has a slow progression: most patients experience advanced deterioration more than 15 years after the clinical onset. Progressive cerebellar ataxia is the core feature, being the only clinical symptom in about 20% of these patients. Therefore, *FMR1* DNA testing could be indicated in individuals aged ≥50 years with unexplained cerebellar ataxia [5]. Parkinsonism has been reported in some patients with FXTAS, but most of them lack rest tremor and the severe rigidity characteristic of idiopathic Parkinson disease [5]. Rare, atypical manifestations, including involuntary movements with generalized chorea, have been described [6]. However, usually, the core clinical features of FXTAS (typically gait ataxia, intention tremor, and cognitive impairment) [7] are always present and not paroxysmal (i.e., “fixed” as opposed to episodic) [1]. In this perspective review, after a brief presentation of the atypical case of an Italian woman with FXTAS who had several paroxysmal episodes, suggestive of acute cerebellar and/or brainstem dysfunction (see Figure 1), we focus our attention on the clinical and preclinical features of FXTAS in women. We searched PubMed at the end of January 2022 for all the articles about “FXTAS” (search term), and we reviewed the 607 abstracts in the English language, to identify relevant publications (see References).

FXTAS is one of the more common single-gene forms of late-onset ataxia and tremor [8]. The molecular mechanisms of the disease include increased *FMR1* mRNA production and toxicity [9]. It is commonly seen as a slowly progressive disease and it is included among the “fixed” forms of ataxia, as opposed to the episodic ataxias which are characterized by recurrent, discrete episodes of neurological dysfunction [10]. Even though paroxysmal movement disorders have been reported in several genetic ataxias (e.g., autosomal recessive spastic ataxia of Charlevoix-Saguenay, or ARSACS) [11], they are not typically present in FXTAS. Nonetheless, non-epileptic episodes of staring and behavioral arrest were reported in children with known fragile X syndrome [12]. FXS is allelic to FXTAS and is due to larger trinucleotide expansions (>200 CGG repeats), leading to a more severe neurodevelopmental disorder. A detailed retrospective study on 19 patients with FXS who underwent electroencephalogram (EEG) revealed that 13 (68%) patients had episodes of staring and behavioral arrest with no EEG correlate, indicating non-epileptic events. One of these patients also had eye-rolling and nystagmus [12]. True epilepsy was rare [12]. Even if complete clinical information is not available, the case with episodic nystagmus might appear as a paroxysmal episode of cerebellar and/or brainstem dysfunction.

In the patient briefly presented in Figure 1, FXTAS was determined to be caused by an expansion of 91 CGG repeats. She had several clinically relevant neuroimaging changes, with brainstem pathology that might explain the paroxysmal episodes of neurological dysfunction, mimicking vertebrobasilar stroke. Repeated CT and MRI scans with angiographic reconstructions and repeated diffusion MRI studies excluded vascular lesions. Furthermore, these episodes were too long to be transient ischemic attacks or epileptic crises; the repeatedly normal EEG outcomes confirm this latter hypothesis. This is the first case where FXTAS shows these phenotypical features. In such cases, a cause–effect relationship may be difficult to be proven. Further research should aim at understanding the mechanisms of this type of paroxysmal episode in FXS and FXTAS.

FXTAS in females has a broad spectrum of symptoms, ranging from slowly progressive to relatively severe diseases with their onset in mid-adulthood or later in life [13]. Proper diagnosis of FXTAS in terms of the identification of expanded *FMR1* alleles is critical to perform appropriate genetic counseling of the extended family [1]. Therefore, a better definition of the clinical picture is fundamental.

We are aware that the association of FXTAS and the paroxysmal episodes observed in our patient may represent a casual coincidence, but our investigations did not permit us to identify any other factors explaining these episodes. In our opinion, reporting such cases to the medical community is fundamental to increase the recognition of the possible and very rare features of a rare disorder.

## 2. “Prodromal” FXTAS

Fragile X premutation carriers are at increased risk for FXTAS. However, the factors which may be able to predict the onset and progression of the disease are unknown.

Premutation carriers without FXTAS may have increased finger tap, hand movement, and rapid alternating movement, both in speed and amplitude, than non-carriers [14]. A cohort study performed on 73 males (48 with the premutation and 25 well-matched controls) did not show significant differences between premutation carriers and controls in executive function tests. However, the premutation carriers had significantly slower manual movements and longer reaction times than controls, and slower movements were observed among the older carriers with a higher number of CGG repeats [14].

A functional MRI study suggested that the 16 *FMR1* premutation carriers of both sexes (including five with FXTAS) who were assessed had deficient visual feedback processing and a reduced cerebellar modulation of corrective motor commands. Therefore, the alterations of the cerebellar-cortical networks during sensorimotor behavior may represent a “prodromal” feature associated with FXTAS degeneration [15].

After this general presentation on both sexes, the next paragraph will be focused on the clinical and preclinical features of FXTAS in women.

## 3. FXTAS in Women

FXTAS in women may have a more complex picture than in men if we consider that: (i) FXTAS features in female carriers can be milder and the penetrance lower; (ii) female premutation carriers can manifest other clinical features, including primary ovarian insufficiency; (iii) women have two X chromosomes, and the non-mutated X may act as a genetic modifying factor; (iv) one of the two X chromosomes can be epigenetically silenced by the DNA methylation; (v) premutation alleles in the X chromosome are typically (but not invariably) unmethylated. [16].

For these reasons, female FXTAS and male FXTAS have a different symptomatic spectrum, and studies on the fragile X premutation should be conducted separately on women or men.

Female carriers of the *FMR1* premutation are characterized by primary ovarian insufficiency and psychiatric issues. A recent cohort study on female premutation carriers reported that these carriers have a neurological phenotype overlapping with the phenotype seen in FXTAS. High rates of neuropathy and tandem gait abnormalities were observed in these studies, that included only patients with the milder symptoms of the FXTAS spectrum [17].

A recent study on 53 females with FXTAS (mean age 67 years) showed a wide range of clinical signs and symptom progression. The imaging results showed the typical ”Middle Cerebellar Peduncle Sign” in only six patients, whereas intensity abnormalities in the splenium of the corpus callosum and the cerebral deep white matter and brainstem were more common. The rate of psychiatric disorders, especially depression, was higher than in the general population [18].

Furthermore, premutations in *FMR1* are the most common monogenic cause of premature ovarian insufficiency, even if the mechanisms that contribute to this pathology are still unclear. This premature menopause (now defined as FXPOI) occurs in about 20% of *FMR1* premutation carriers compared with approximately 1% in the general population [19].

## 4. Biomarkers, FXTAS Pathophysiology, Mitochondrial Dysfunction, and Potential Drug Targets

In contrast to the “full” mutation (>200 repeats), which leads to transcriptional silencing of the *FMR1* gene, in FXTAS there is not a substantial reduction in protein levels. However, in “premutated” subjects there is a marked increase in *FMR1* mRNA. This increase has been postulated to be toxic through various mechanisms [1]. The pathological hallmarks include ventricular enlargement, focal white matter lesions, ubiquitinated intranuclear inclusion bodies, patches of astrogliosis, and excessive iron accumulation [1].

The presence of ubiquitin-positive intranuclear inclusions in neurons and astrocytes is the major criterion for the pathological diagnosis. A recent neuropathological study revealed intranuclear inclusions in the endothelial cells of capillaries and an increased number of cerebral microbleeds in the brains of subjects with FXTAS, suggesting that microangiopathy may be a pathologic feature of FXTAS. Furthermore, an association between the number of capillaries containing amyloid β in the cerebral cortex and disease progression was observed [20].

Several recent studies were focused on the identification of potential biomarkers for FXTAS. A study of longitudinal metabolic profiling reported that the sub-pathways of lipid metabolism involved in mitochondrial bioenergetics were altered in FXTAS [21]. Increased levels of the proteins involved in acute phase response signaling, liver X receptor/ retinoid X receptor (LXR/RXR) activation, and farnesoid X receptor (FXR)/RXR activation were identified in another study on the cerebrospinal fluid (CSF) proteome. Furthermore, changes in proteins associated with other neurodegenerative disorders (i.e., amyloid-like protein 2, contactin-1, afamin, cell adhesion molecule 4, NPC intracellular cholesterol transporter 2, and cathepsin B), as well as significant changes of several apolipoproteins, were identified [22]. Another study on 42 females revealed that cognitive impairment strongly correlated with both mitochondrial dysfunction and CGG repeat length. A combined multi-omics approach identified a down-regulation of RNA and mRNA metabolism and translation, carbon and protein metabolism, unfolded protein response, and up-regulation of glycolysis and antioxidant response [23].

Various mitochondrial dysfunctions were reported in models of FXTAS; however, the underlying molecular mechanisms are still unclear [24]. For instance, altered mitochondrial function was reported in cells carrying a premutation or unmethylated full mutation of the *FMR1* gene [25]. Some mitochondrial DNA (mtDNA) polymorphisms may represent genetic modifying factors for FXTAS; thus, individuals with FXTAS may accumulate higher rates of heteroplasmic mtDNA variants [26]. The reduced number of mtDNA copies in the brain may also be related to FXTAS progression [27]. Moreover, MRI findings could correlate with peripheral mitochondrial bioenergetics [28]. Further research on the role of mitochondrial dysfunction in FXTAS is needed.

A recent study [29] on primary dermal fibroblasts from FXTAS-affected carriers showed an increased immune response. This was probably the result of a new epitope produced by the oxidation of the accumulated, unfolded proteins, RNA and DNA. The down-regulation of proteostasis and mitochondrial function, limiting the cellular repair capacity, underscores this phenomenon. RNA metabolism was up-regulated, whereas its translation was down-regulated [29]. The phytochemical drug, sulforaphane, had beneficial actions on pathways related with brain function, bioenergetics, unfolded protein response, proteosome, antioxidant defenses, and iron metabolism in FXTAS fibroblasts. Sulforaphane improved all the aspects of the mitochondrial function in fibroblasts, especially in coupling between electron transport and ATP production [29]. Additionally, the treatment with allopregnanolone may have a positive impact on GABA metabolism, oxidative stress, and mitochondrial dysfunction in males with FXTAS [30]. Allopregnanolone is the neuroactive metabolite of progesterone; the actions of sex hormones on mitochondria might explain, maybe partially, the different clinical characteristics of males and females with a fragile X premutation [31]. Considering that, to date, no effective treatment is available, randomized clinical trials are strongly needed to verify the efficacy of allopregnanolone for treating FXTAS [32].

## 5. Management of FXTAS

The therapeutic options for treating subjects with FXTAS are only symptomatic. When the clinical picture resembles essential tremor, beta-blockers may be used. Levo-Dopa may be beneficial in patients with parkinsonism. Psychiatric symptoms, including anxiety or depression, should be treated with standard therapies and psychological support. Medications, like memantine, that can slow cognitive decline may be useful, but further studies are needed [32]. Recently, a male FXTAS patient with chorea treated with tetrabenazine experienced thrombocytopenia and agranulocytosis [33], suggesting that these patients should be carefully monitored.

Future trials should focus on the possibility of blocking the translation of CGG-expanded RNA into the “toxic” polyglycine-containing protein, by using small molecules [34] or short antisense oligonucleotide steric blockers [35].

## 6. Conclusions

It is important to consider FXTAS in patients with undiagnosed ataxia (and/or tremor), regardless of gender [36,37]. The clinical presentation of FXTAS is variable and can include tremor, cerebellar ataxia, neuropathy, and/or cognitive decline; radiological features include brain atrophy and white matter disease in specific brain regions. The phenotypical outcomes of FXTAS require the expression of the abnormal *FMR1* mRNA, but the exact links between mRNA and the clinical features are still unclear [1]. Furthermore, it must be noted that the penetrance in males with 70 or less CGG repeats is very low [38].

FXS and FXTAS are usually found in the same families and often multiple individuals with one of these conditions can be found. Cascade testing for these mutations is necessary to allow all the members of the affected families to get the correct diagnosis [1]. Even if female premutation carriers develop FXTAS at lower rates than males, they are at risk for FXPOI, which represents the most heritable form of premature menopause or early ovarian failure. In addition, female premutation carriers may have higher rates of psychiatric symptoms including anxiety, attention deficit hyperactivity disorder, depression, insomnia, chronic fatigue, and chronic pain [39]. The incidence and severity of these symptoms need to be investigated in ad-hoc longitudinal studies. Furthermore, the MRI “Middle Cerebellar Peduncle Sign” is not often seen in women, however, MRI scans reveal increased signals in the splenium of the corpus callosum and the pons [39]. Therefore, female FXTAS and male FXTAS may represent different disorders, and studies on the fragile X premutation should focus separately on women or men.

Subtle motor impairments correlating with cognitive and, mainly, executive deficits may occur in female premutation carriers not meeting the diagnostic criteria for FXTAS [40]. Among those who do not have other comorbid diagnoses, women who have CGG repeats at the lower end of the premutation range may be at greater risk for ataxia and parkinsonism than their age-matched peers, although their overall risk of developing such clinical features is low [41]. Furthermore, women with the “premutation” who have a family history of FXTAS may be at increased risk for this neurodegenerative disease [42], suggesting that other genetic factors may also have a role. However, these preliminary data require confirmation by larger studies.

A very recent study showed that gait ataxia and kinetic tremor progressed more rapidly in males than females with FXTAS. In contrast, psychiatric symptoms significantly progressed only in females [43].

Females with FXTAS have a broad spectrum of symptoms, ranging from slowly progressive, relatively severe diseases starting in mid-adulthood, to mild cases beginning later in life. Atypical features (e.g., dystonia) can be found in women [44] (Table 1).

Hopefully, a better understanding of the molecular processes involved in the mediation of the polymorphic features of FXTAS will lead to more specific and effective therapies for this, still elusive, disorder. This may also include the use of progesterone derivatives (such as the neurosteroid, allopregnanolone) [45], but further studies are needed.

The criteria for defining a subject as an FXTAS patient or “premutation carrier” are not always clear in the literature. Furthermore, the term “premutation” may be misleading because this is a real and true pathogenic mutation, even if its phenotypes are polymorphous and its penetrance incomplete. There is a strong need for a biologically and clinically more rational nomenclature, based on the results of large multicenter studies. We believe that new, correct, genotype and phenotype definitions may contribute to a more homogeneous categorization of patients, useful for performing new effective experimental and real-world studies.

## Figures and Tables

**Table 1:** Fragile X-associated tremor/ataxia syndrome (FXTAS) in males and females. The relative frequencies are indicative (specifically designed epidemiological studies are not available). Refs., selected references (see bibliography).

	Males	Females	Refs.
Tremor, ataxia, cerebellar signs	very common	very common	[1,2]
Gonadal failure	very rare (not reported)	very common	[2]
Cognitive impairment	very common	common (milder than in males)	[1,4]
Psychiatric issues	common	very common	[43]
Atypical features (e.g., dystonia)	very rare	rare	[44]
Middle Cerebellar Peduncle Sign	very common	rare	[18,39]
